# MicroRNA 135a Suppresses Lymph Node Metastasis through Down-Regulation of ROCK1 in Early Gastric Cancer

**DOI:** 10.1371/journal.pone.0085205

**Published:** 2014-01-21

**Authors:** Ji-Young Shin, Young-Il Kim, Soo-Jeong Cho, Mi Kyung Lee, Myeong-Cherl Kook, Jun Ho Lee, Sang Soo Lee, Hassan Ashktorab, Duane T. Smoot, Keun Won Ryu, Young-Woo Kim, Il Ju Choi

**Affiliations:** 1 Center for Gastric Cancer, National Cancer Center, Goyang, Korea; 2 Department of life Science and Biotechnology, Paichai University, Daejeon, Korea; 3 Departments of Medicine, Howard University, Washington, District of Columbia, United States of America; National Cancer Center, Japan

## Abstract

MicroRNAs (miRNAs) play a critical role in gastric cancer progression and metastasis. This study investigated the role of miRNA-135a in early gastric cancer (EGC) including lymph node (LN) metastasis. We examined the correlation between miRNA-135a expression and clinical outcomes in 59 patients who underwent surgery for EGC. Using gastric cancer cell lines, we performed functional and target gene analyses. miRNA-135a expression was down-regulated in 33.9% of patients. These patients showed a significantly more advanced stage (TNM stage≥IB, 35.0% vs. 12.8%, p = 0.045) and higher rate of LN metastasis (30.0% vs. 5.1%, p = 0.014) than those with up-regulation of miRNA-135a expression. In a multivariate analysis, down-regulation of miRNA-135a was an independent risk factor for LN metastasis (adjusted odds ratio, 8.04; 95% confidence interval, 1.08–59.81; p = 0.042). Functional analyses using gastric cancer cell lines showed that miRNA-135a suppressed cell viability, epithelial-mesenchymal transition, cell invasion, and migration. ROCK1 was a target of miRNA-135a and its expression was inversely correlated to that of miRNA-135a. ROCK1 expression was significantly increased in EGC patients with LN metastasis than in those without LN metastasis. Our results confirm the tumor-suppressive role of miRNA-135a, and demonstrate its role in LN metastasis in EGC. miRNA-135a and its target gene *ROCK1* may be novel therapeutic and prognostic targets for EGC.

## Introduction

Gastric cancer still remains the second most common cause of cancer mortality worldwide, despite decreasing incidence and mortality rates in developed countries [1].

In areas, including Korea and Japan, where screening for gastric cancer is performed widely, early detection is often possible. Because an increased rate of early detection of gastric cancer can lead to an improved prognosis, interests in improving patients' quality of life and utilizing minimally invasive treatments have increased. In patients with gastric cancer, lymph node (LN) metastasis is one of the most important prognostic factors, and the overall incidence of a LN metastasis in early gastric cancer (EGC) ranges from 5 to 20% [2]–[4].

Gastrectomy with LN dissection is regarded as the standard treatment for gastric cancer; however, 80 to 95% of patients with EGC do not require a LN dissection if the gastric cancer is completely excised by less invasive treatments such as endoscopic resection [5]–[7] or minimally invasive surgery (*e.g.*, simple wedge resection or sentinel LN navigation surgery) [8]–[10]. Although studies of biologic prognostic and predictive markers for LN metastasis in EGC have been conducted, no predictive tools exist for nodal metastasis of EGC actually.

Several studies have reported that depth of invasion, tumor size, and lymphatic invasion are associated with LN metastasis in EGC [4], [11]. However, most of these factors have been determined after final pathological examination of resected tissues, which is not useful in determining treatment strategy.

MicroRNAs (miRNAs) are small non-protein coding RNAs. Studies have proven that alterations in expression of miRNAs contributes to the pathogenesis of cancers including those of gastrointestinal origin [12]–[15].

miRNAs have been also been shown to be useful in differentiating cancer and normal tissues [16], [17]. In addition, miRNAs are expressed differentially in each stage of gastric cancer carcinogenesis [18]. miRNA-27 [19] and the ZEB1-regulated miRNA-200 family [20] have shown to be related to stage of epithelial-mesenchymal transition (EMT) in gastric cancer. Therefore, miRNAs may be associated with the process of LN metastasis in EGC. Among miRNAs, miRNA-135a has been shown to be a tumor suppressive miRNA, and its expression is down-regulated in several cancers including renal cell carcinoma [21] and lymphoma [22]. In turn, over-expression or restoration of miRNA-135a promotes apoptosis and suppresses proliferation of tumor cells through its target genes c-Myc [21] and JAK2 [22], respectively. In an *in vitro* study, miRNA-135a suppressed migratory and invasive activities of gastric cancer cells [23]. However, the tumor suppressive role of miRNA-135a in gastric cancer is unclear.

The aim of this study was to investigate the correlation between miRNA-135a expression and clinical outcomes in EGC patients including LN metastasis.

## Materials and Methods

### Human tissue samples and clinical data

Fifty-nine adult patients diagnosed with EGC at the National Cancer Center, Korea, from January 2011 to April 2013 were prospectively included in this study. These patients were treated with an open or laparoscopy-assisted gastrectomy with D1+ or more LN dissection. The extent of LN dissection followed the recommendations of the Japanese Gastric Cancer Association [24]. The stage after surgery was evaluated according to the 7^th^ edition of American Joint Committee on Cancer TNM staging system [25]. Human tissues including both gastric cancer and matched normal tissues from each patient were immediately frozen in liquid nitrogen and stored at −80°C. From the review of medical charts, clinicopathologic characteristics including age, sex, *Helicobacter pylori* status, tumor characteristics, stage, and status of LN metastasis were obtained and analyzed. Informed written consent was obtained from all patients and the study was approved by the Institutional Review Board of the National Cancer Center, Korea (NCCNCS-11-445).

### Cell culture and transfection

**Figure 1:**
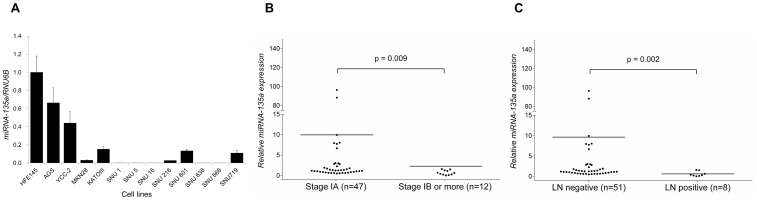
miRNA-135a expression patterns in gastric cancer cell lines and human early gastric cancer tissues. (A) miRNA-135a expression, examined by real-time PCR analysis, is down-regulated in gastric cancer cells compared with normal gastric mucosa cells (HFE 145 cells). (B) Relative miRNA-135a expression of tumor tissue in reference to normal counterparts by real-time PCR analysis according to patient stage.^*^ Patients with more advanced cancer stage (stage IB or greater) show significantly down-regulated miRNA-135a expression levels in tumor tissues compared to levels in patients with less advanced stage (stage IA) cancer (mean 9.97 vs. 2.30, p = 0.009). (C) Relative miRNA-135a expression of tumor tissue in reference to normal counterparts in real-time PCR analysis according to LN metastasis status. Patients with LN metastasis show significantly down-regulated miRNA-135a expression levels in tumor tissues (when compared to corresponding normal tissues) than patients without LN metastasis (mean 9.63 vs. 0.61, p = 0.002). *7^th^ American Joint Committee on Cancer TNM staging.

The human normal gastric epithelial cell line HFE145 [26], [27] was a gift from Dr Hassan Ashktorab and Duane T. Smoot (Howard University, Washington, USA) and commercially available gastric cancer cell lines AGS, YCC2, MKN28, KATOIII, SNU1, SNU5, SNU16, SNU216, SNU601, SNU638, SNU668, and SNU719 were obtained from the Korea Cell Line Bank (KCLB, Seoul, Korea). All cell lines were maintained in RPMI 1640 media containing 10% fetal bovine serum (FBS) and 1% antibiotics (Invitrogen, Carlsbad, CA, USA). Among the gastric cancer cell lines, MKN28 and SNU668 cell lines were selected because they rarely express miRNA-135a, and YCC and KATOIII cell lines were selected because they have a substantial baseline expression of miRNA-135a (Figure 1A). Transfections with miRNA-135a mimics (mirVana™ miRNA mimics, Ambion, Austin, TX, USA), an miRNA-135a inhibitor (mirVana™ miRNA inhibitor, Ambion, Austin, TX, USA), and ROCK1 siRNA (5′-UGAUGCAAAGAUUGUACUC-3′; UGBioneer, Seoul, Korea) in SNU668, YCC2, MKN28, and KATOIII cell lines were performed using Lipofectamine RNAiMAX and Lipofectamine 2000 reagents (Invitrogen, Carlsbad, CA, USA) according to the manufacturer's instructions. MKN28, SNU668, YCC2, and KATOIII cell lines were harvested two days after transfection, and various analyses were performed.

### RNA extraction and miRNAs quantification

Total RNA was isolated from 13 cell lines and 59 gastric cancer patients' tissues, respectively, by using TRIzol (Invitrogen, Carlsbad, CA, USA) as per the manufacturer's protocol. The TaqMan universal PCR master mix reagents kit (Applied Biosystems, Tokyo, Japan) was used. Amplification and detection were performed using a 7900HT Sequence Detection System (Applied Biosystems, Tokyo, Japan) with 40 cycles of denaturation at 95°C (15 seconds) and annealing/extension at 60°C (60 seconds). This was preceded by reverse transcription at 50°C for 30 minutes and denaturation at 95°C for 10 minutes. To quantitate mature miRNAs, TaqMan MicroRNA Assays kits (Applied Biosystems, Tokyo, Japan) were used for detection of miRNA-135a and a control miRNA (RNU6B).

### Western blot analysis

Protein was extracted from 13 cell lines and 59 EGC patients' tissue samples by using RIPA buffer (Biosesang, Seoul, Korea) including a protease inhibitor cocktail (Sigma, St. Louis, MO, USA) according to the manufacturer's protocol. Next, Western blotting was performed using anti-ROCK1 and anti-β-actin (Santa Cruz Biotechnology, Santa Cruz, CA, USA) antibodies. Signals were detected with an ECL prime kit (GE healthcare, Piscataway, NJ, USA) as per the manufacturer's instructions.

### Cell proliferation assays

Cell proliferation was measured using an MTT assay. Cells were plated in 96-well culture plates (3×10^3^ cells per well) and transfected with miRNA-135a mimics after two days of incubation. Subsequently, 200 µl of MTT (0.5 mg/ml, Sigma, St. Louis, MO, USA) were added to each well. After four hours of additional incubation, MTT solutions were discarded, 200 µl of DMSO (Amresco, Solon, OH, USA) were added to each well, and the plate was shaken gently. Absorbance was measured on an ELISA reader (Moecular Devices, Sunnyvale CA, USA) at a test wavelength of 570 nm.

### Cell cycle analysis

Cells were plated in 60π culture plates and transfected with miRNA-135a mimics or a miRNA mimics negative control (Mimics-NC). These cells were harvested by trypsinization and fixed in 70% ethanol for at least two hours at −20°C. Cell pellets were washed with cold phosphate-buffered saline and incubated for 30 minutes at room temperature in PI/RNase Staining Buffer (BD Biosciences, San Diego, CA, USA). After staining, samples were analyzed with a FACScan flow cytometer (BD Biosciences, San Jose, CA, USA) and 10,000 events were collected per sample. Data from flow cytometry were analyzed using Cell Quest software (Becton Dickinson, San Jose, CA, USA).

### Trans-filter migration and invasion assays

The cells were transfected with miRNA-135a mimics, miRNA-135a inhibitors, ROCK1 siRNA, and ROCK1 siRNA negative control (scRNA) for one day and then transferred to the upper chamber of the transwell plate coated with 0.5 mg/ml collagen type I (BD Bioscience, Bedford, MA, USA) and a 1∶15 dilution of Matrigel (BD Bioscience, Bedford, MA, USA). RPMI 1640 with 10% FBS and 1% antibiotics (Gibco BRL, Grand Island, NY, USA) was added to the lower chamber and the plate was incubation for 20 hours. Migrating and invading cells were quantified using hematoxylin and eosin staining.

### The luciferase reporter plasmid constructions

The wild type 3′ UTR of ROCK1 containing binding sites for miRNA-135a was amplified using gDNA from SNU668 cells as a template. The correlated mutant constructs were created by mutating the seed regions of the miRNA-135 binding sites. Both wild type and mutant 3′ UTR were cloned in downstream of the luciferase gene in a pGL3 control vector. The constructs were verified by sequencing.

### Luciferase assay

Luciferase assays were performed in SNU668 and YCC2 cells. SNU668 and YCC2 cells were transfected with each of the plasmids (empty vector [MOCK], ROCK1 3′ UTR wild type [WT], and ROCK1 3′ UTR mutant [MUT], as an miRNA-135a binding site) together with miRNA-135a mimics, miRNA-135a inhibitors, and negative control RNA in 24-well plates. Two days after transfection, cells were harvested and lysed. Luciferase assays were performed on the lysates by using a luciferase assay kit (Promega, Madison, WI, USA). Luciferase activity was normalized to β-galactosidase.

### Immunohistochemical staining

Immunohistochemical (IHC) staining was performed for representative 20 cases from ROCK1 low and high expression groups. Antigen retrieval was done with heat treatment for 30 min in pH 8.0 Tris-EDTA buffer (CC1, Ventana Medical System, Tucson, AZ, USA) at 95°C. Tissue slides were incubated at 42°C for 30 min with anti-ROCK1 mAb (ab134181, clone EPR638Y, rabbit monoclonal, 1∶5,000; Abcam, Cambridge, MA, USA). Negative controls were used identically with nonimmunized rabbit IgG. Results were interpreted by an experienced pathologist (MC Kook).

### Statistical analysis

Scale data are given as mean±standard deviation (SD). Chi-square or Fisher's exact tests and the Mann-Whitney U test were used to compare categorical variables and continuous variables, respectively. The Pearson correlation coefficient was used for comparing the gene expression pattern between miRNA-135a and ROCK1. A multivariate logistic regression model was used to determine independent factors associated with LN metastasis. Covariates that were significant using chi-square or Fisher's exact tests (*P*<0.05) were entered into the logistic regression model. All data were analyzed using STATA 12.1 (Stata Corp, College Station, TX, USA). A *P* value of less than 0.05 was considered statistically significant.

## Results

### miRNA-135a expression levels are associated with progression and LN metastasis in EGC

A previous study showed that miRNA-135a expression is down-regulated in gastric cancer cell lines [23]. By using qRT-PCR, we also found that miRNA-135a expression is down-regulated in various gastric cancer cell lines compared with that in a normal gastric epithelial cell line (Figure 1A).

However, the correlation between miRNA-135a expression and clinical outcomes in gastric cancer has not been investigated. Therefore, we measured the miRNA-135a expression levels in primary gastric cancer and their corresponding levels in normal tissues from 59 patients with EGC. Down- and up-regulation of genes in tumor tissues with respect to normal tissues were defined as the ratio of tumor to normal tissue gene expression levels of <1.0 and >1.0, respectively.

Conversely to the result of *in vitro* experiment, only 20 (33.9%) of 59 patients with EGC exhibited down-regulation of miRNA-135a expression. However, these patients showed significantly a more advanced stage (TNM stage≥IB, 35.0% vs. 12.8%, p = 0.045; crude odd ratio [OR], 2.11; 95% confidence interval [CI], 1.08–4.10) and a higher rate of LN metastasis (30.0% vs. 5.1%, p = 0.014; crude OR, 2.73; 95% CI, 1.50–4.98) than those with up-regulation of miRNA-135a expression (Table S1). Sensitivity and specificity of miRNA-135a expression status for prediction of LN metastasis were 75.0% and 72.5%, respectively. In addition, patients with more advanced stage (Figure 1B) or LN metastasis (Figure 1C) showed a significantly lower ratio of tumor to normal miRNA-135a expression levels than those with stage IA or without LN metastasis. A multivariate logistic regression analysis also showed that miRNA-135a down-regulation was independently associated with LN metastasis (adjusted OR, 8.04; 95% CI, 1.08–59.81; p = 0.042; Table 1). Collectively, these findings suggest that down-regulation of miRNA-135a expression may be associated with gastric cancer progression including LN metastasis.

**Table 1:** Factors associated with lymph node metastasis in patients with early gastric cancer.

Variable, n (%)	LN metastasis		p	Multivariate analysis		p
	No (n = 51)	Yes (n = 8)		Odds ratio	95% CI	
Age ≥65 years	20 (39.2)	5 (62.5)	0.265			
Male sex	39 (36.5)	6 (75.0)	0.928			
Smoking	18 (35.3)	1 (12.5)	0.416			
Alcohol	31 (60.8)	4 (50.0)	0.704			
BMI ≥25 kg/m^2^	17 (33.3)	5 (62.2)	0.135			
Family history of gastric cancer	10 (19.6)	0 (0)	0.329			
H. pylori infection	38 (74.5)	6 (75.0)	0.976			
Tumor characteristics						
Tumor size ≥5 cm	17 (33.3)	6 (75.0)	0.047	7.01	0.90–54.92	0.064
Tumor location			0.896			
Upper 1/3	11 (21.6)	2 (25.0)				
Middle 1/3	18 (35.3)	2 (25.0)				
Lower 1/3	22 (43.1)	4 (50.0)				
Undifferentiated histology*	34 (66.7)	6 (75.0)	0.639			
Diffuse type	17 (33.3)	2 (25.0)	0.874			
Lymphovascular invasion	8 (15.7)	5 (62.5)	0.010	11.28	1.56–81.57	0.016
miRNA-135a down-regulation	14 (27.5)	6 (75.0)	0.014	8.04	1.08–59.81	0.042

LN, lymph node; BMI, body mass index.

* Undifferentiated histology includes poorly differentiated adenocarcinoma or signet ring cell carcinoma.

### miRNA-135a suppresses gastric cancer cell viability, EMT, migration, and invasion

**Figure 2:**
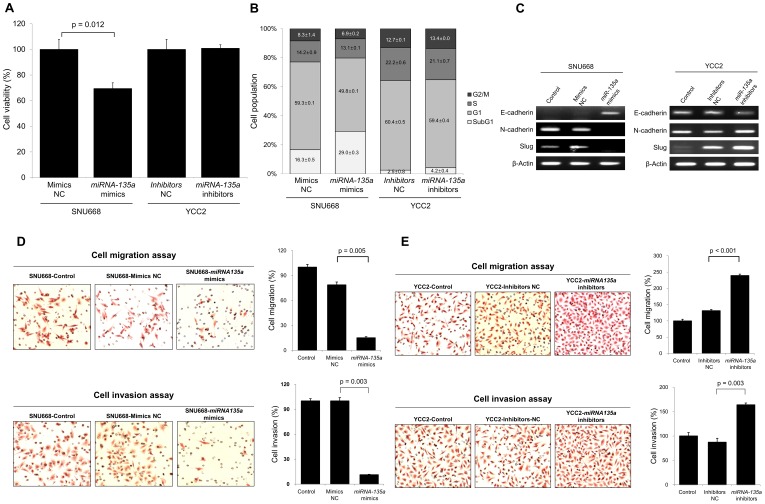
miRNA-135a suppresses cell viability and inhibits EMT factors, cell invasion, and migration in gastric cancer cells. (A) Up-regulation of miRNA-135a induced by miRNA-135a mimics suppresses gastric cancer cell viability in SNU668 cell line, a cell line which rarely expresses miRNA-135a (p = 0.012), whereas miRNA-135a inhibitors rarely affect cell viability in YCC2 cell line, a cell line which substantially expresses miRNA-135a. (B) Up-regulation of miRNA-135a by miRNA-135a mimics increases the subdiploid fraction of DNA, as determined by flow cytometry analysis, suggesting increased apoptosis in SNU668 cells. (C) In the presence of miRNA-135a mimics, RT-PCR analysis shows that SNU668 cells have increased mRNA expression of E-cadherin and suppress mRNA expression of both N-cadherin and Slug. Conversely, in the presence of miRNA-135a inhibitors, YCC2 cells show decreased mRNA expression of E-cadherin and increased mRNA expression of both N-cadherin and Slug in RT-PCR analysis. (D) Overexpression of miRNA-135a using miRNA-135a mimics significantly suppresses migration and invasion of SNU668 cells. (E) Inhibition of miRNA-135a using miRNA-135a inhibitors significantly increases migratory and invasive cells of the YCC2 cells. Results shown are the means ± SD of at least three experiments. NC, negative control.

To further investigate the association between miRNA-135a and clinical outcomes, we performed functional assays for cell viability, cell cycle, EMT, migration, and invasion in gastric cancer cell lines. Overexpression of miRNA-135a by miRNA-135a mimics significantly suppressed cell viability (Figure 2A) and increased the subdiploid fraction of SNU668 cells, a cell line that rarely expresses miRNA-135a (Figure 2B), suggesting the increased apoptosis of gastric cancer cells. However, suppression of miRNA-135a by miRNA-135a inhibitors induced neither an increase in cell viability nor a subdiploid fraction decrease in YCC2 cells, a cell line that expresses substantial miRNA-135a (Figure 2A and 2B). We also investigated the association between miRNA-135a expression and EMT in gastric cancer cells, because miRNA-135a down-regulation was an independent risk factor for LN metastasis in EGC patients (Table 1). Increased expression of miRNA-135a was associated with suppression of EMT and showed an increase in the expression of E-cadherin and the suppression of both N-cadherin and Slug in SNU688 cells. In contrast, inhibition of miRNA-135a expression resulted in activation of EMT including the suppression of E-cadherin expression and the up-regulation of both N-cadherin and Slug expression in YCC2 cells (Figure 2C). In the assessment for functional consequences, overexpression of miRNA-135a by miRNA-135a mimics significantly suppressed the migration and invasion abilities of SNU668 gastric cancer cells (Figure 2D). Conversely, blockade of miRNA-135a by miRNA-135a inhibitors significantly increased migration and invasion of YCC2 gastric cancer cells (Figure 2E). Overall, our results suggested that miRNA-135a is a tumor suppressive regulator for gastric cancer proliferation and metastasis.

### ROCK1 is a target gene of miRNA-135a in gastric cancer

**Figure 3:**
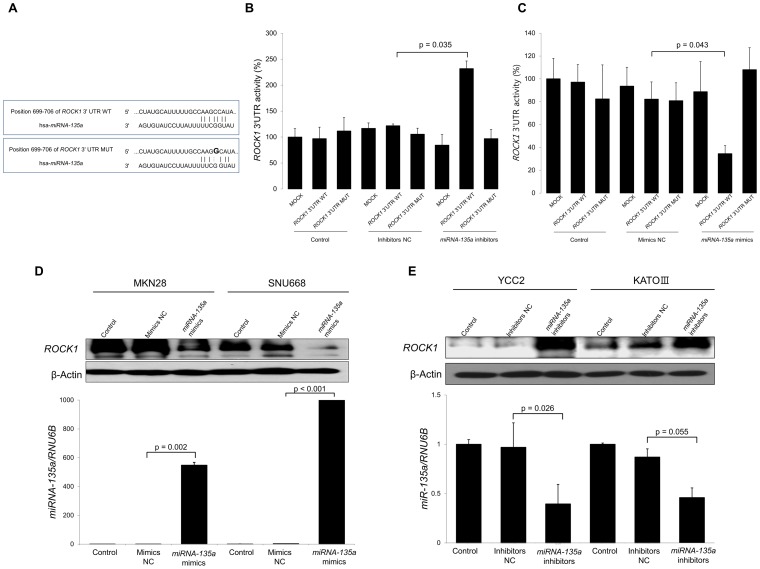
ROCK1 is a direct target of miRNA-135a in gastric cancer cells. (A) The 3′ UTR sequence of ROCK1 has one possible binding site for miRNA-135a, as determined by TargetScan analysis. The 3′ UTR constructs for ROCK1 WT and MUT are shown. (B) In the presence of miRNA-135a inhibitors, relative luciferase activity of ROCK1 3′ UTR WT is significantly increased compared to ROCK1 3′ UTR MUT in SNU668 cells (p = 0.035). (C) On the contrary, in the presence of miRNA-135a mimics, the relative luciferase activity of ROCK1 3′ UTR WT is significantly decreased (p = 0.043), whereas the relative luciferase activity of ROCK1 3′ UTR MUT is not decreased in the YCC2 cell line. (D) Western blot analysis shows that overexpression of miRNA-135a using miRNA-135a mimics suppresses ROCK1 protein expression in MKN28 and SNU688 gastric cancer cells, which are cell lines that rarely express miRNA-135a. (E) Inhibition of miRNA-135a associated with increased ROCK1 protein expression in YCC2 and KATO III gastric cancer cells, which are cell lines that substantially express miRNA-135a. Results shown are the means ± SD of at least three experiments. WT, wild type; MUT, mutant; NC, negative control.

To elucidate the mechanisms underlying the effects of miRNA-135a on clinical outcomes, we searched putative targets by using the TargetScan 6.2 database. Of 718 conserved targets, we selected targets with highest score whose down-regulation might result in increased cancer cell proliferation, migration and invasion. We found that ROCK1 might be a target gene of miRNA-135a because inhibition or suppression of ROCK1 has been reported to promote apoptosis and to suppress migration, invasion, and proliferation of cancer cells including those of gastric cancer [28]–[30]. TargetScan analysis revealed that the 3′UTR sequence of ROCK1 has one possible binding site for miRNA-135a (Figure 3A). To confirm whether ROCK1 is a target of miRNA-135a, a luciferase assay was performed. The luciferase 3′UTR constructs described in methods section were co-transfected into SNU688 cells with miRNA-135a mimics and into YCC2 cells with miRNA-135a inhibitors. We also constructed ROCK1 3′UTR MUT by point mutation C→G in the possible binding site (Figure 3A). The relative luciferase activity of the ROCK1 3′UTR WT was significantly increased in the presence of miRNA-135a inhibitors (Figure 3B). Conversely, this activity was significantly decreased in the presence of miRNA-135a mimics (Figure 3C). In gastric cancer cell lines, miRNA-135a and ROCK1 showed an opposing expression pattern. Western blot analysis showed that ROCK1 expression was down-regulated in the presence of miRNA-135a mimics, and up-regulated in the presence of miRNA-135a inhibitors (Figure 3D and 3E). Taken together, these results indicate that miRNA-135a suppresses ROCK1 expression by directly targeting its 3′UTR.

### Expression of ROCK1 is inversely correlated to that of miRNA-135a in patients with EGC

**Figure 4:**
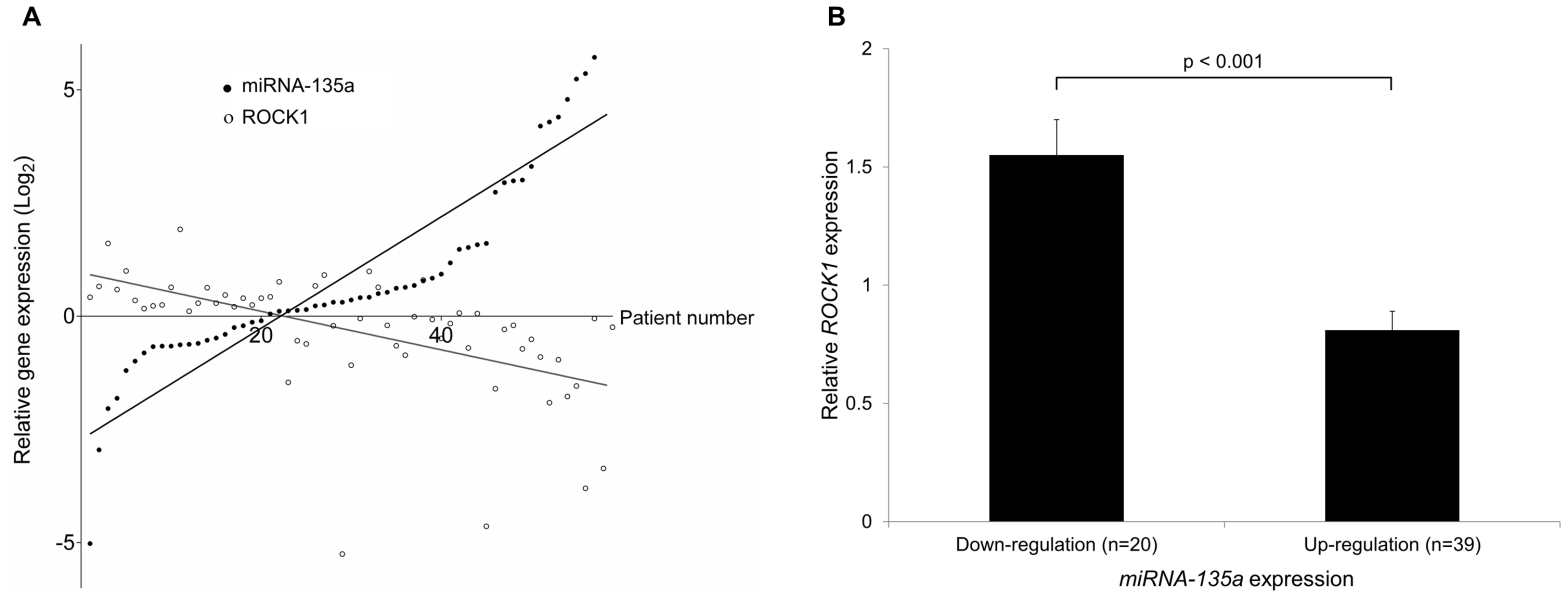
miRNA-135a expression is inversely correlated to ROCK1 protein expression in human early gastric cancer tissue. (A) miRNA-135a expression was measured by real-time PCR analysis. The ROCK1 protein level was evaluated using western blot analysis and quantified using densitometric method. In 59 patients with early gastric cancer, expression of ROCK1 shows a significantly negative correlation with that of miRNA-135a (r = −0.697, p<0.001). (B) Western blot analysis shows that patients with down-regulated miRNA-135a expression have significantly higher ROCK1 protein expression in tumor tissues compared to expression in corresponding normal tissues (p<0.001).

Analysis of 59 patients with EGC showed that expression levels of ROCK1 had a significantly negative correlation with those of miRNA-135a (r = −0.697, p<0.001; Figure 4A). In addition, patients with up-regulation of miRNA-135a expression had significantly lower levels of ROCK1 protein expression than those with down-regulation of miRNA-135a (mean tumor to normal ratio, 0.81 vs. 1.55, p<0.001; Figure 4B).

### ROCK1 is associated with LN metastasis in EGC and is involved in gastric cancer cell migration and invasion suppressed by miRNA-135a

**Figure 5:**
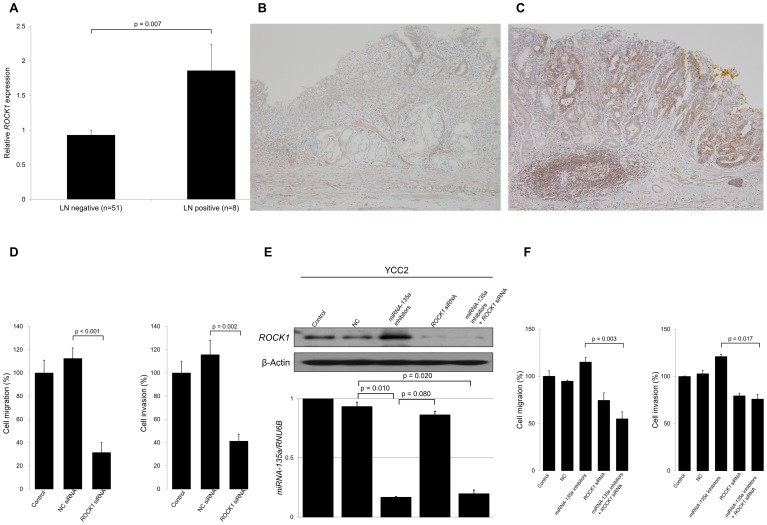
The association between ROCK1 expression and clinical outcomes. (A) Western blot analysis of 59 early gastric cancer patients shows that patients with LN metastasis have significantly higher ROCK1 protein expression than patients without LN metastasis (p = 0.007). Representative microscopic images of tumor sections with immunohistochemical staining (original magnification ×100) show that (B) early gastric cancer patients without LN metastasis have no ROCK1 expression in tumor tissues, but (C) those with LN metastasis have strong ROCK1 expression within the nuclear membrane and cytoplasm of tumor tissues. (D) ROCK1 siRNA significantly suppresses migration (p<0.001) and invasion (p = 0.002) in SNU668 gastric cancer cells. (E) ROCK1 protein expression is increased by treatment with miRNA-135a inhibitors and decreased by treatment with ROCK1 siRNA, and is associated with an increased miRNA-135a level in YCC2 gastric cancer cells, suggesting that ROCK1 protein expression patterns are inversely correlated with those of miRNA-135a expression. (F) When YCC2 cells are treated with miRNA-135a inhibitors, cell invasion and migration are similar or even increased compared to controls. ROCK1 siRNA or combined ROCK1 siRNA and miRNA-135a inhibitors decrease cell migration (p = 0.003) and invasion (p = 0.017). These suggest that ROCK1 offsets the suppressive effects of both migration and invasion that is induced by miRNA-135a. Results shown are the means ± SD of at least three experiments. LN, lymph node; NC, negative control.

To further evaluate the association between ROCK1 expression and clinical outcomes in EGC, we analyzed the patterns of ROCK1 expression in reference to the LN metastasis status of included patients. Patients with LN metastasis had a significantly higher expression of ROCK1 protein than those without LN metastasis (mean tumor to normal ratio, 1.86 vs. 0.93, p = 0.007; Figure 5A). Furthermore, levels of ROCK1 IHC staining were similar to those of ROCK1 expression in western blot analysis (*i.e.*, patients without LN metastasis had weak IHC staining patterns [Figure 5B] but those with LN metastasis had strong positive IHC staining [Figure 5C]).

Finally, to investigate whether the suppressed migration and invasion abilities conferred by the miRNA-135a up-regulation observed in gastric cancer cell lines were the result of ROCK1 up-regulation, we performed migration and invasion assays using gastric cancer cell lines transfected with specific ROCK1 siRNA. ROCK1 siRNA suppressed migratory and invasive activities in SNU668 cells (Figure 5D). In YCC2 cells co-transfected with miRNA-135a inhibitors and ROCK1 siRNA (Figure 5E), migration and invasion assays showed that inhibition of ROCK1 by siRNA significantly attenuated the enhancing migratory and invasive effects of miRNA-135a down-regulation by miRNA-135a inhibitors (Figure 5F).

Collectively, these results suggest that ROCK1 is associated with LN metastasis in EGC patients, and that this results from increased ROCK1 expression in the context of miRNA-135a down-regulation. Therefore, ROCK1 may be involved in miRNA-135a-suppressed gastric cancer metastasis.

## Discussion

In the present study, we found that miRNA-135a had tumor suppressive roles in gastric cancer. In patients with EGC, down-regulation of miRNA-135a was an independent risk factor for LN metastasis. In gastric cancer cells, up-regulated miRNA-135a suppressed gastric carcinogenesis by suppressing cell proliferation, EMT, and metastasis. Furthermore, we also identified a new target gene of miRNA-135a, ROCK1, which was associated with LN metastasis in gastric cancer.

In patients with EGC, evaluation of LN metastasis before treatment is an important step in the determination of treatment strategies. Less invasive treatments such as endoscopic resection [5]–[7] or sentinel LN navigation surgery [9], [10] may be possible in EGC patients without LN metastasis. These treatment approaches have improved patients' quality of life in respect to late complications associated with standard surgical treatments including dumping syndrome, weight loss, and reflux-related symptoms [31]. Therefore, more specific and sensitive biomarkers are required to predict the status of LN metastasis in patients with EGC. In the present study, miRNA-135a down-regulation was an independent factor for LN metastasis, and prediction value of miRNA-135a expression status was relatively high with a sensitivity 75% and a specificity 73%. Although further validation studies are needed, these results showed that measurement of miRNA-135a levels prior to determining the treatment plan for EGC patients could be a useful test for predicting LN status.

Interestingly, miRNA-135a has been reported to be tumor suppressive and oncogenic. Overexpression of miRNA-135a has been associated with suppression of tumor proliferation and growth in renal cell carcinoma [21] and with increased apoptosis and better disease-free survival in lymphoma [22]. In contrast, miRNA-135a was involved in colorectal cancer progression [32] and in increased breast cell migration and invasion [33]. In the present study, miRNA-135a was shown to be a tumor suppressive miRNA, which is in agreement with the result of a previous study [23]. Specially, the higher expression of miRNA-135a suppressed several steps of gastric carcinogenesis including proliferation, migration, and invasion. Furthermore, we also found that miRNA-135a might suppress EMT, which has reported to be associated with the initiation of the multistep process of carcinogenesis and promotion of metastasis [34], [35]. Prior to the present study, the association between miRNA-135a and EMT has not been investigated, although previous studies have shown that miRNAs such as miRNA-27 [19], the miRNA-200 family [20], miRNA-7 [36], and miRNA-1228 [37] suppress EMT through their own target genes in gastric cancer. Taken together, miRNA-135a is a tumor suppressive miRNA in gastric cancer.

A previous study analyzing 353 human gastric cancer tissues showed that expression patterns of miRNAs are diverse and that some miRNAs were associated with progression and prognosis in gastric cancer [14]. In the study, miRNA-135a was classified as an oncogenic miRNA in gastric cancer based on the results of DNA microarray analyses. However, in a recent [23] and our study, miRNA-135a acted as a tumor suppressor despite the high rate of patients with miRNA-135a up-regulation. Although many patients (66%) had up-regulation of miRNA-135a similar to the result of a previous study [14], these patients showed a significantly less advanced stage and a lower rate of LN metastasis. Furthermore, additional *in vitro* studies also showed that a higher expression of miRNA-135a was associated with suppression of gastric carcinogenesis. These results suggest that expression patterns of miRNAs might be different from clinical outcomes, and therefore, to confirm the exact roles of specific miRNAs, further studies are needed.

ROCK1 is a member of the Rho-associated serine/threonine kinase family, which functions as a central regulator of actin cytoskeleton organization and dynamics [23], [38]. ROCK1 has a diverse range of functions in tumorigenesis including migration, invasion, and metastasis [28]. ROCK1 is a target of several miRNAs in various cancers including miRNA-584 in renal cell carcinoma [39], miRNA-335 in neuroblastoma [40], and miRNA-146a in prostate cancer [41]. In gastric cancer, ROCK1 has been positively correlated with LN metastasis and TNM stage [42], and is involved in miRNA-148a-induced suppression of gastric cancer cell migration and invasion [30]. Further, ROCK1 inhibitor promoted apoptosis in gastric cancer cells [29]. In the present study, we found that ROCK1 is a target of miRNA-135a in gastric cancer and its expression is positively associated with LN metastasis in EGC patients. *In vitro* experiments also showed that ROCK1 is involved in gastric cancer cell motility and invasion. These results suggest that ROCK1 may be a new therapeutic target with the ability to inhibit gastric cancer metastasis.

In conclusion, we have identified miRNA-135a as a new prognostic biomarker for LN metastasis in patients with EGC and demonstrated that miRNA-135a suppressed gastric carcinogenesis (*i.e.*, proliferation, EMT, and metastasis in gastric cancer cell lines) by targeting ROCK1. Although these findings require validation in other independent patients groups, they suggest that miRNA-135a and its target gene ROCK1 may be a new biomarker and therapeutic target for gastric cancer with LN metastasis.

## Supporting Information

Table S1 **Clinicopathologic characteristics of patients with early gastric cancer according miRNA-135a expression status.**
(DOC)
