# Supplementary material for: MicroRNA 135a Suppresses Lymph Node Metastasis through Down-Regulation of ROCK1 in Early Gastric Cancer
Source: PLoS One. 2014 Jan 21;9(1):e85205. doi: 10.1371/journal.pone.0085205 (PMC3897422; doi:10.1371/journal.pone.0085205)
Supplement: Table S1 — Clinicopathologic characteristics of patients with early gastric cancer according miRNA-135a expression status. (DOC) [file pone.0085205.s001.doc]

Table S1. Clinicopathologic characteristics of patients with early gastric cancer according miRNA-135a expression status

|  | miRNA-135a expression^*^ | | p |
| --- | --- | --- | --- |
|  | Down-regulation  (n= 20) | Up-regulation  (n=39) |  |
| Age, years | 61.6±10.7 | 61.5±11.6 | 0.997 |
| Sex, n (%) |  |  | 0.869 |
| Male | 15 (75.0) | 30 (76.9) |  |
| Female | 5 (25.0) | 9 (23.1) |  |
| Current smoking, n (%) | 7 (35.0) | 12 (30.8) | 0.742 |
| Alcohol ingestion, n (%) | 14 (70.0) | 21 (53.8) | 0.232 |
| Body mass index, kg/m^2^ | 24.0±3.1 | 24.1±3.7 | 0.903 |
| Family history of gastric cancer, n (%) | 5 (25.0) | 5 (12.8) | 0.283 |
| H. pylori positive, n (%) | 15 (75.0) | 29 (74.4) | 0.957 |
| Tumor characteristics |  |  |  |
| Size, cm | 4.69±1.75 | 4.67±2.31 | 0.975 |
| Tumor location, n (%) |  |  | 0.053 |
| Upper 1/3 | 7 (35.0) | 6 (15.4) |  |
| Middle 1/3 | 3 (15.0) | 17 (43.6) |  |
| Lower 1/3 | 10 (50.0) | 16 (41.0) |  |
| Differentiation^†^, n (%) |  |  | 0.151 |
| Differentiated | 4 (20.0) | 15 (38.5) |  |
| Undifferentiated | 16 (80.0) | 24 (61.5) |  |
| Lauren classification, n (%) |  |  | 0.169 |
| Intestinal type | 10 (50.0) | 22 (56.4) |  |
| Diffuse type | 5 (25.0) | 14 (35.9) |  |
| Mixed type | 5 (25.0) | 3 (7.7) |  |
| Post-operative stage^‡^, n (%) |  |  | 0.045 |
| IA | 13 (65.0) | 34 (87.2) |  |
| IB or more advanced | 7 (35.0) | 5 (12.8) |  |
| Lymphovascular invasion, n (%) | 6 (30.0) | 7 (17.9) | 0.332 |
| Lymph node metastasis, n (%) | 6 (30.0) | 2 (5.1) | 0.014 |
| Numerical variables were expressed by mean±standard deviation.  *Relative expression level in tumor tissue compared with paired normal tissue  †Differentiated histology includes papillary, well and moderately differentiated adenocarcinoma and undifferentiated histology includes poorly differentiated adenocarcinoma or signet ring cell carcinoma.  ‡7^th^ American Joint Committee on Cancer TNM stage | | | |
